# Heat Stress Trends in Regions of Intensive Turkey Production in Germany—A Challenge in Times of Climate Change

**DOI:** 10.3390/ani14010072

**Published:** 2023-12-24

**Authors:** Björn Sake, Nina Volkmann, Nicole Kemper, Jochen Schulz

**Affiliations:** Institute for Animal Hygiene, Animal Welfare and Farm Animal Behavior, University of Veterinary Medicine Hannover, Foundation, 30173 Hannover, Germany; nina.volkmann@tiho-hannover.de (N.V.); nicole.kemper@tiho-hannover.de (N.K.); jochen.schulz@tiho-hannover.de (J.S.)

**Keywords:** climate change, animal welfare, Turkeys, enthalpy, THI, heat stress, global warming, poultry

## Abstract

**Simple Summary:**

This study investigated heat loads determined using the parameters enthalpy and temperature–humidity index (THI). For this purpose, weather station data were used, and specified threshold exceedance in German districts with intensive turkey production was analyzed. Over a period of 50 years, increases in both parameters were observed in all districts. Most of the trends in terms of exceeding the thresholds, and thus towards heat stress, were statistically significant. Heat stress in turkeys should, therefore, not be underestimated, especially when considering ongoing global warming.

**Abstract:**

This study analyzed trends of enthalpy and the temperature–humidity index (THI) over a period of 50 years in outer air, which lead to severe heat stress in turkeys. Weather station data from 15 German districts with high densities of turkey production were used to investigate the heat input into the barns. Therefore, the parameters of enthalpy and THI with specified thresholds were used for heat stress assessment. Trends in extreme weather situations where these thresholds were exceeded were analyzed and tested for significance using the Mann–Kendall test. In all districts, the heat load increased between 1973 and 2022 for both parameters. Statistically significant heat stress trends were found in 9 of the 15 districts for enthalpy and 14 out of 15 districts for THI. Thus, the established THI thresholds seem to be more sensitive for the detection of heat stress than the chosen enthalpy values. As heat stress is an important issue and a rising concern in times of climate change, farmers and constructors of farm animal facilities should take this into account in future sustainable work.

## 1. Introduction

Climate change and the associated global warming may affect the performance and health of farm animals [1]. Meteorologists predict that the earth will become increasingly warmer and, therefore, heat stress on farm animals may likewise increase [2]. In regions with rising temperatures, if no appropriate cooling measures are introduced, animals may increasingly leave their thermal neutral zone, also known as the comfort zone [3]. Consequences could be a reduced animal performance by poorer feed conversion [4,5,6], changes in intestinal microflora [5], reduced milk yield [4], and poorer fertility [4,7], for instance. Another economic aspect is the negatively affected meat quality of pig and poultry meat when animals grow under heat stress [8,9,10]. In the case of poultry, feathering and missing sweat glands make birds particularly susceptible to heat stress compared to other monogastric animals [5].

Turkey production plays an important role in Germany and explicitly in the federal state of Lower Saxony [11]. In 2021, about 32% of the global production with 1853 thousand tonnes of carcass weight was produced in the European Union (EU) [12]. Of all EU countries, Germany was the largest producer with 363 thousand tonnes of slaughter weight in 2021 [12]. Turkeys in Germany are typically kept in naturally ventilated barns with additional fans inside to create wind chill effects [13], which also may be equipped with fogging or sprinkler systems. Especially at the end of fattening periods, high enthalpy values can lead to heat stress within the flocks and birds react with decreasing food intake as well as an increasing water consumption and respiration rate that may appear as exaggerated panting [14]. Furthermore, their body temperature may increase, and turkeys can become dehydrated at different, high temperature–humidity combinations. This can occur in all age groups [15,16]. Behavioral changes such as lethargy, less activity, and seeking for cooler areas could be observed. If no counter measures are taken and heat stress persists, productivity is affected negatively, and the mortality rate can increase [17,18,19,20].

The effects of global warming vary regionally. While regions are growing warmer, it is not yet clear how changes in ocean currents will have an impact on some continental regions [21]. Therefore, trend analyses can help in assessing local climate change and its potential impact on livestock.

However, focusing on temperature alone is not sufficient to assess heat stress in animals. The enthalpy (H) of moist air is a key factor when animals regulate their temperature. The higher the humidity at the same temperature, the higher H. Birds normally lose about half of their body heat through evaporation as they breathe [22]. Nonetheless, higher relative humidity (RH) reduces their possibility of evaporation. At high RH and high temperatures, birds have problems reducing excess heat. In extreme situations, such as a prolonged time at 85% RH and 35 °C, for instance, turkeys can even die [22]. In Germany, for instance, at H values ≥ 67 kJ/kg in the outer air, farmers are encouraged to control the climate on their farms and to consider measures to reduce heat stress in their flocks [23]. Above that threshold, farmers should provide ventilation to ensure adequate air exchange in the barn [24].

To assess heat stress on farm animals, H values, or the energy content of the air calculated as the specific enthalpy (kJ/kg) as well as the temperature–humidity index (THI) are both used. For turkey production, such H values and different THIs considering heat stress are available.

THIs were created for many species and were classified to values representing ‘no heat stress’ or ‘extreme heat stress’ [25]. Moraes et al. [26] mentioned that turkeys suffer from severe discomfort when THI reaches values of 81.

However, a retrospective calculation of enthalpy and THI frequencies may provide information about heat stress trends in local regions. This could be useful information for farmers concerned with future planning and preventive measures for their animals concerning heat stress.

The aim of this study was to analyze trends of heat stress in German regions of dense turkey populations using enthalpy and THI values calculated from weather data of the last 50 years. Thresholds of severe heat stress were considered to highlight critical weather situations, which also might have a negative health impact on the birds in the barns. Possible consequences for future turkey production are discussed.

## 2. Materials and Methods

### 2.1. Weather Station Data

**Figure 1:**
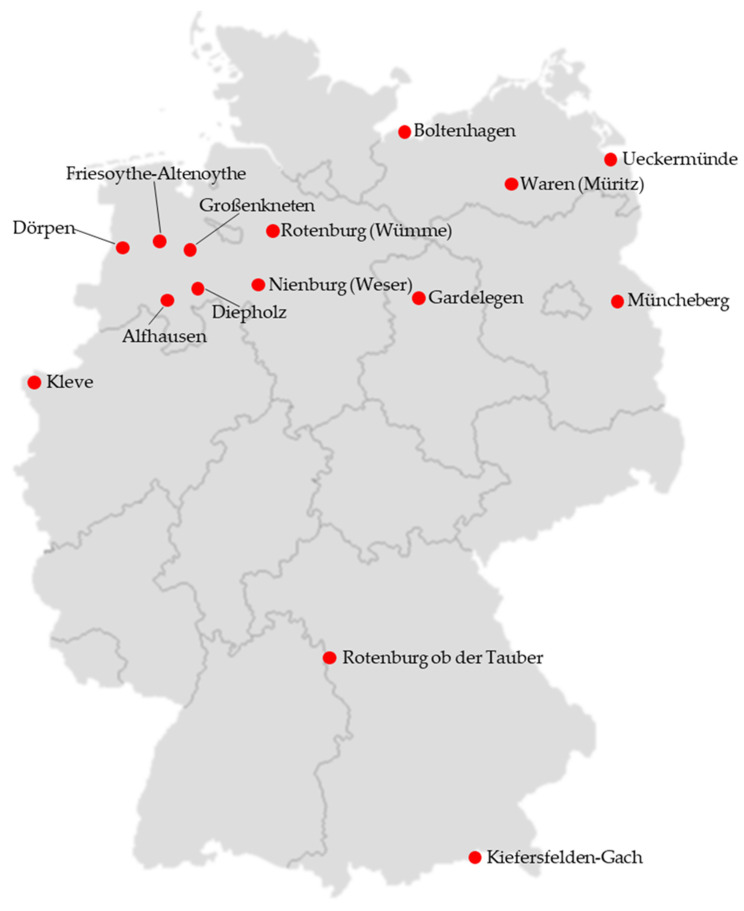
Map of Germany with its 16 federal states. The locations of the weather stations in the 15 different districts with large turkey populations are marked with reds dots. The map was created with the help of mixmaps.de [27].

Data from 15 weather stations from the years 1973 to 2022 provided by the German Weather Service ‘Deutscher Wetterdienst (DWD)’ concerning 15 German districts (Table S1) with particularly intensive turkey production [11] were analyzed and are shown in Figure 1. These are predominantly in Northern Germany, particularly in Lower Saxony.

As Germany belongs to the temperate climate zone, heat stress usually occurs from late spring to early fall. Thus, in the present study, yearly data from May 1st to September 30th were considered for analysis. Data contained the daily maximum air temperature (°C), the daily mean relative humidity (RH, %), and the daily enthalpy maximum (kJ/kg) calculated by the DWD. The H values provided were used for the analysis, while the THI was calculated using the specified temperature and humidity values.

### 2.2. Heat Stress Classification

For poultry, the DWD classified the calculated enthalpy values to heat stress classes, which are shown in Table 1 [28].

An enthalpy value of 67 kJ/kg ambient air was chosen as the threshold, as according to the DWD, severe or extreme heat stress can be expected in poultry houses above this level.

In addition to the enthalpy classification by DWD, THI limits were considered to classify turkeys’ heat stress according to Moraes et al. [26] (Table 1), taking into account the following Equation (1) (firstly presented by Thom [29] and modified by Buffington et al. [30]):

THI = 0.8 Tdb + RH (Tdb − 14.3)/100 + 46.3(1)

with:

- Tdb = air dry-bulb temperature (°C);
- RH = relative humidity of air (%).

As the study by Moraes et al. [26] described the heat stress classes as ‘bird comfort’, their formulations have likewise been adopted in Table 1.

For trend analyses, the classes from ‘severe heat stress’ and ‘extreme heat stress’ concerning enthalpy values ≥ 67 kJ/kg were set as heat stress thresholds and were compared to THI classes of ‘severe discomfort’ and ‘life threatening’ with values ≥ 81. For data processing, frequencies in days over the thresholds of the chosen heat stress classes in the complete data set were determined (Excel 2016, Microsoft Corporation, Redmond, WA, USA), and at each location exceedances of these thresholds of both parameters were counted for each year.

### 2.3. Statistical Analysis

Statistical analysis was performed using RStudio version 4.2.1 [31] and the package ‘trend’ [32]. Data had been tested for normal distribution and autocorrelation previously. Heat stress trend lines for each location and for all locations were created and their gradients and gradient equations were specified. Furthermore, during the 50 analyzed years, the additional number of days on which enthalpy (≥67 kJ/kg) and THI (≥81) exceeded the threshold was calculated for each location (Table 2). Average values from all weather stations were calculated by averaging the frequencies, which represents the number of days with exceedance, in the individual years. These frequencies were then used to create a graph and a trend line with a trend line equation. The values 1 and 50 (for the beginning and end of the study period) were used in this trend line equation. The determined values are shown in Table 3. Finally, significant increases in the heat stress trend during the 50 years were analyzed for each location using a (modified) Mann–Kendall test. A level of significance set at *p* < 0.05 was determined.

## 3. Results

Enthalpy values between 11.8 and 83.6 kJ/kg were recorded at all weather stations analyzed during the 50-year period. The THI values varied between 39.97 and 91.84. For descriptive statistics, trend lines were drawn for both parameters and all weather stations. For all locations the gradient of the trend lines was positive, indicating a general increase in high enthalpy values and high THIs over the previous 50 years, as the gradient indicated an increase from year to year and therefore an annual increase. The results of the gradient of the trend line for each location are shown in Table S2. Gradient values for enthalpy ranged from 0.0069 in Rotenburg (Wümme) to 0.0647 in Kleve. For the THI, the values ranged between 0.0378 in Boltenhagen and 0.2602 in Kiefersfelden-Gach (Table S2). Generally, the frequencies and variations in the THI trend lines were higher compared to those given enthalpy thresholds.

**Figure 2:**
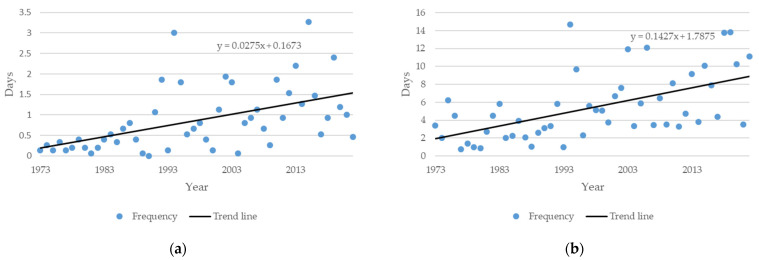
Average frequencies in days of exceeded thresholds concerning (**a**) enthalpy values (≥67 kJ/kg) and (**b**) temperature–humidity index (THI) values (≥81) over all locations in the analyzed years from 1973 to 2022. The gradient of the trend line can be read from the equation by looking at the factor in front of the variable. The years are plotted on the *x*-axis. The trend line equation was created for the period of 50 years and therefore also calculates with the values from 1 to 50, which are used as variable in the equation.

The average values of the number of days exceeding the enthalpy threshold for severe and extreme heat stress of ≥67 kJ/kg are shown in Figure 2a, and those of the average THI number of days exceeding the threshold of values ≥ 81, representing ‘severe discomfort’ and ‘life threatening’ (Table 1), are shown in Figure 2b. Both trend lines were positive.

The results of the increases in days for the entire study period are listed in Table 2. The additional number of days that were added to the base level due to the increases that were recorded exceeding the enthalpy thresholds ranged from 0.4 days in Rotenburg (Wümme) to 3.2 days in Kleve, for instance. The additional number of days exceeding the enthalpy thresholds ranged from 0.4 days in Rotenburg (Wümme) to 3.2 days in Kleve, for instance. Regarding the additional number of days where the THI thresholds were exceeded for the analyzed period, these ranged from 1.9 days in Boltenhagen to 13.0 days in Kiefersfelden-Gach (Table 2).

The value calculated with the trend line equation where the threshold value of 67 kJ/kg was exceeded amounted to 0.19 days for enthalpy for all locations in Germany in 1973 and averagely 1.54 days in 2022 (Table 3). Concerning the THI, the average value calculated with the trend line equation where the threshold was exceeded for all locations increased from averagely 1.93 days in 1973 to 8.92 days in 2022 (Table 3).

The results of the (modified) Mann–Kendall test analyzing significant trends during the 50-year period for each site are shown in Table 4. For the parameter enthalpy, 9 out of 15 trends were significant. Except for one location (Rotenburg (Wümme) *p* = 0.094), all increases for THI were significant (*p* < 0.05) (Table 4).

## 4. Discussion

Climate change may not only increase temperatures but also the enthalpy of air, which affect the welfare, productivity and health of farm animals. The present study investigated the trends of high enthalpy values and high THIs in regions of intense turkey production in Germany.

High density of commercial turkey farms in particular regions combined with the difficulty of obtaining weather data from some districts during the last decades limited the number of weather stations, which could be used for the study. Thus, this study analyzed data of an unequal distribution of selected weather stations in Germany.

The chosen thresholds for enthalpy (≥67 kJ/kg) and THI (≥81) of outer air were suspected of causing severe heat stress and severe discomfort, respectively. The effects of such high values on poultry can be manifold and might result in poorer meat quality [8,10], lower productivity [9,10,19], or an increased mortality rate [9,18,19]. However, although heat stress might already occur at lower values in turkey flocks, this study aimed to show how more extreme weather situations might impair turkeys’ health and welfare due to an ongoing climate change in future. Significant increases in trend were found in 9 of the 15 regions for high enthalpy values and in 14 of 15 regions for high THIs, respectively.

Concerning the average number of occurrences, THI values showed more days of exceedance per year up until 2022 (8.9 days, Table 3) than the exceedance of enthalpy values (1.5 days, Table 3).

It is assumed that the threshold set by Moraes et al. [26] is more sensitive and more likely to indicate an onset of severe discomfort due to the different application of the values. In the regions examined in this study, humidity and temperature data revealed THI values that exceeded the threshold earlier compared to enthalpy values. This is not necessarily applicable for other regions because temperature and humidity combinations may differ. However, the choice of thresholds may impact prevention measures. For instance, high mortality rates in poultry barns were reported from veterinary offices during the heat waves in Germany in 1992 and 1994. Measures to reduce the heat in barns were not taken into account, not sufficient or the conduction of counter-measures was too late [33]. Observations and measurements by veterinarians revealed that outside enthalpies higher than 60 kJ/kg led to lethal enthalpy values in poultry barns. In a case in the district Diepholz in northern Germany in 1994 for instance, 7.5% of broilers died at enthalpy values of 60.6 kJ/kg outside and 73.7 kJ/kg in the barn, respectively [34]. In this year, an average of 3 days with enthalpies ≥ 67 kJ/kg (Figure 2a) and an average of 22 days with THI values ≥ 81 (Figure 2b) occurred. From empirical observations within the two heat wave periods in Germany it was concluded that sufficient ventilation in poultry barns prevent from death due to heat when outside enthalpy values are below 67 kJ/kg. However, birds suffer from heat stress below 67 kJ/kg and even if no irreversible pathological findings are detectable, productivity can be affected. Therefore, the THI ≥ 81 as a threshold to consider additional measures to reduce heat stress would probably contribute to fewer affected birds in the later fattening period.

Moraes et al. [26] compiled specific thresholds for poultry and classification of THI into ranges of bird comfort or discomfort. The trends in the districts can vary greatly; for example, in the district Boltenhagen (located at the Baltic Sea), only 1.9 additional days exceeded the THI, while in Kiefersfelden-Gach (located in southern Germany with proximity to the mountains), the additional number of days exceeding the THI amounted to 13.0. Therefore, regional climatic differences can be influenced by geographic location, topography, land use and microclimatic conditions [35]. In addition to time series analysis, the exploration of variability in spatial relationships complemented long-term trend analysis, especially in this study covering a large spatial extent. As demonstrated by Cesca et al. in beef cattle [35], the use of descriptive statistics makes it possible analyze spatial dependence, and classify the degree of data dependence according to thermal comfort indices. Therefore, for studies involving historical series of spatially distributed meteorological data, the joint use of temporal and spatial analyses, which can provide relevant additional information, is recommended. This implies that models may be useful to analyze regional consequences of climate change, or to predict requirements for a successful production of farm animals in future.

Furthermore, it seems that as the number of days with heat stress increases, so does the energy in the outdoor air. For instance, the trend in the district Kleve showed no enthalpy value over equal 67 kJ/kg until 1986. Nevertheless, in the following years (1986–2022), there was a total of 62.0 days exceeding above the specified threshold, and the highest number in any one year was six (6.0 days in 2015, Figure S1).

The used Mann–Kendall test is a robust non-parametric test for time series trends and thus preferably used in studies of hydrologic time series and climatic variations [36,37,38]. Other studies calculated complex weather forecast models to analyze regional trends of climate factors [39,40,41]. However, for Germany, a positive temperature trend was forecast [42]. As temperature is one of the main factors for THIs and enthalpy values, it can be assumed that these parameters will also continue to rise.

Despite current and future efforts to reduce heat stress through management or technical measures, the question remains as to whether production costs, and therefore the costs and losses caused by heat stress, will increase. Necessary measures could include feeding strategies [43], reducing stocking density [16], or supplemental cooling through additional air [44], heat exchangers, etc. However, even if management and cooling can reduce the heat content of the air in the barn, these capacities are limited [45] and livestock farmers should consider whether their systems are able to cope with climate change. For instance, in warm regions, turkey barns are usually equipped with cooling systems such as fogger systems, spray cooling, or pad cooling systems [13]. All systems are based on the principle of evaporation chill and can reduce heat stress in the flock efficiently [22]. However, some requirements and limitations have to be considered. First, it is important that microbiologically uncontaminated water in the system is used because the birds might inhale aerosols. Second, the systems should not damp the litter in the barn due to a potential risk of an increased occurrence of footpad dermatitis. According to Mailyan et al., systems should not be activated or operated when the RH in the barn is >80% [22]. Furthermore, we have to keep in mind that heat stress increases relatively at higher RH, as evaporation is the main process to regulate the birds’ temperature. For instance, a fogging system may decrease according to manufacturer the temperature in a poultry barn by 7 °C when the outer air temperature is 36 °C and the humidity of incoming air is 50% [46]. At the average atmospheric pressure at sea level of 1013.25 hPa, this air contains an enthalpy of 84.5 kJ/kg [47]. Thus, when the air is cooled by a fogging system to 29 °C but simultaneously humidity increases to 85% [46], the enthalpy stays at 84.5 kJ/kg [47]. Therefore, the measure might decrease the temperature, but the enthalpy remains high, and the birds stay stressed. As shown in the trend analyses in the present study, days with higher enthalpy values have already intensified and probably will become more frequent in future. Thus, to avoid additional heat stress situations, other, or additional measures should be taken into account to protect the birds and to maintain their performance, health, and welfare. This could also include calculating and regulating the entire climate in animal houses based on enthalpy values and not only by measuring temperatures. In this context, the management and equipment of the barn play a crucial role. These can not only provide relief, but also create situations that are more comfortable than those outside the barn. The barn provides shade from direct sunlight and cooling from the ventilation systems. Heat stress is therefore not only a problem of indoor housing but also of outdoor housing [25].

Further possibilities to counter increasing heat stress situations due to climate change in future could be the adaptation of breeds. The genetics of the animals and therefore their metabolism and heat production have obviously changed over time. Sensitivity to heat depends on genetics [1], and the occurring breeds experience heat stress in warm regions. Thus, keeping and fattening heat-resistant breeds could be of even greater interest in the future.

Concerning the rapidly growing world population and the ever-increasing challenges associated with climate change, it seems essential to maintain poultry production. This should also apply to other species and other areas of agriculture. Heat stress has a direct impact on food security, animal welfare, and production [20] and obviously represents a challenge for future farm animal production.

## 5. Conclusions

The trend analyses conducted in this study showed an increase in enthalpy and THI values in the investigated German districts. It is assumed that these increases have a negative impact on the animals in the barns if not counteracted. In future, it is expected that housing systems will face greater challenges in coping with heat stress and therefore need to be adapted to meet these challenges. Enthalpy values and THIs should be used in addition to temperature control to assess the climate in the barn and possible countermeasures.

## Figures and Tables

**Table 1:** Heat stress classification for poultry with units of measurement and description referring to the enthalpy values from German Weather Service (DWD) [28] and temperature–humidity index (THI) values according to Moraes et al. [26].

Enthalpy		THI	
Value [kJ/kg]	Description	Value	Description
<50	no heat stress	≤72	absolute comfort
50 to <58	mild heat stress	73 to 76	light discomfort
58 to <67	moderate heat stress	77 to 80	moderate discomfort
67 to <72	severe heat stress	81 to 84	severe discomfort
≥72	extreme heat stress	≥85	life threatening

**Table 2:** Gradient for enthalpy and temperature–humidity index (THI) for each weather station over the whole study period and calculated in days. This gradient indicates the additional days achieved by the annual gradient. For this purpose, the annual gradient was multiplied by the study period of 50 years. The values indicate the difference in days applied at the end of the period compared to the beginning.

Weather Station	Enthalpy [Days]	THI [Days]
Alfhausen	1.25	4.09
Boltenhagen	0.52	1.89
Diepholz	0.68	4.89
Dörpen	1.85	4.93
Friesoythe-Altenoythe	0.47	5.10
Gardelegen	0.48	10.46
Großenkneten	2.81	9.26
Kiefersfelden-Gach	2.86	13.01
Kleve	3.24	8.46
Müncheberg	0.99	11.26
Nienburg	1.47	6.91
Rotenburg (Wümme)	0.35	3.63
Rothenburg ob der Tauber	0.39	11.37
Ueckermünde	2.49	4.94
Waren (Müritz)	1.07	6.83
Average	1.375	7.14

**Table 3:** Number of days over all locations when enthalpy (≥67 kJ/kg) and temperature–humidity index THI (≥81) thresholds were exceeded in the specified year, calculated using the trend line equation.

Year	Enthalpy	THI
1973	0.19	1.93
2022	1.54	8.92

**Table 4:** Results of the analysis of the gradient (Mann–Kendall’s Tau and *p*-values) of the heat stress trends for each weather station in terms of enthalpy (≥67) and temperature–humidity index (THI, ≥81), calculated using the Mann–Kendall test.

Weather Station	Enthalpy ≥ 67		THI ≥ 81	
	Tau	*p*-Value	Tau	*p*-Value
Alfhausen	0.270	0.016	0.220	0.030
Boltenhagen	0.174	0.134	0.290	0.008
Diepholz	0.175	0.119	0.210	0.039
Dörpen	0.379	<0.001	0.275	0.007
Friesoythe-Altenoythe	0.166	0.129	0.228	0.025
Gardelegen	0.098	0.392	0.448	<0.001
Großenkneten	0.559	<0.001	0.474	<0.001
Kiefersfelden-Gach	0.341	0.001	0.440	<0.001
Kleve	0.512	<0.001	0.404	<0.001
Müncheberg	0.304	0.006	0.494	<0.001
Nienburg	0.003	0.010	0.333	<0.001
Rotenburg (Wümme)	0.108	0.339	0.170	0.094
Rothenburg ob der Tauber	0.111	0.333	0.483	<0.001
Ueckermünde	0.352	0.001	0.465	<0.001
Waren (Müritz)	0.300	0.007	0.417	<0.001
Average	0.425	<0.001	0.426	<0.001

## Data Availability

The datasets used and/or analyzed during the current study are available from the corresponding author on reasonable request.

## Supplementary Materials

The following supporting information can be downloaded at: https://www.mdpi.com/article/10.3390/ani14010072/s1, Figure S1: Number of days of exceeded thresholds concerning enthalpy values (≥67 kJ/kg) in Kleve (from 1973 to 2022); Table S1: Location of weather stations (data provided by the German weather service (Deutscher Wetterdienst). The number of turkeys and farms are listed for each district; Table S2: Gradients of the trend line equation for the enthalpy values and temperature–humidity index (THI) for each weather station. Gradients indicate the average increase from year to year as the gradient corresponds to the annual increase in days.
