# Supplementary material for: Heat Stress Trends in Regions of Intensive Turkey Production in Germany—A Challenge in Times of Climate Change
Source: Animals (Basel). 2023 Dec 24;14(1):72. doi: 10.3390/ani14010072 (PMC10778477; doi:10.3390/ani14010072)
Supplement: Supplementary file 1 [file animals-14-00072-s001.zip › Figure S1.pdf]

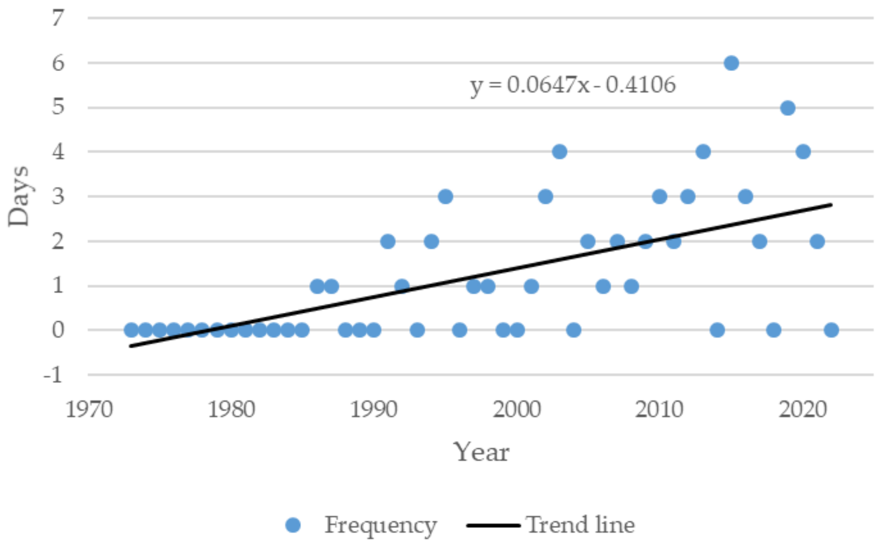

Figure S1. Number of days of exceeded threshold concerning enthalpy values (≥ 67 kJ/kg) in Kleve (from 1973 to 2022).
