# Supplementary material for: Heat Stress Trends in Regions of Intensive Turkey Production in Germany—A Challenge in Times of Climate Change
Source: Animals (Basel). 2023 Dec 24;14(1):72. doi: 10.3390/ani14010072 (PMC10778477; doi:10.3390/ani14010072)
Supplement: Supplementary file 1 [file animals-14-00072-s001.zip › Table S1.pdf]

Table S1. Location of weather stations (data provided by the German weather service (Deutscher Wetterdienst). The number of turkeys and farms are listed for each district [11].

District	Weather Station	Number of turkeys	Turkey farms
Osnabrück	Alfhausen	151,356	13
Nordwestmecklenburg	Boltenhagen	24,710	4
Diepholz	Diepholz	206,577	21
Emsland	Dörpen	548,803	34
Cloppenburg	Friesoythe-Altenoythe	2,043,811	107
Altmarkkreis Salzwedel	Gardelegen	348,005	12
Oldenburg	Großenkneten	915,344	55
Rosenheim	Kiefersfelden-Gach	144,633	23
Kleve	Kleve	485,338	30
Märkisch-Oderland	Müncheberg	178,191	11
Nienburg (Weser)	Nienburg	139,407	8
Rotenburg (Wümme)	Rotenburg (Wümme)	257,659	17
Ansbach	Rothenburg ob der Tauber	156,683	30
Vorpommern-Greifswald	Ueckermünde	212,944	9
Mecklenburgische Seenplatte	Waren (Müritz)	81,349	10
	Total	5,894,810	384
