# Supplementary material for: Heat Stress Trends in Regions of Intensive Turkey Production in Germany—A Challenge in Times of Climate Change
Source: Animals (Basel). 2023 Dec 24;14(1):72. doi: 10.3390/ani14010072 (PMC10778477; doi:10.3390/ani14010072)
Supplement: Supplementary file 1 [file animals-14-00072-s001.zip › Table S2.pdf]

Table S2. Gradients of the trend line equation for the enthalpy values and temperature-humidity index (THI) for each weather station. Gradients indicate the average increase from year to year as the gradient corresponds to the annual increase in days.

Weather Station	Enthalpy	THI
Alfhausen	0.0249	0.0818
Boltenhagen	0.0104	0.0378
Diepholz	0.0136	0.0978
Dörpen	0.0369	0.0985
Friesoythe-Altenoythe	0.0093	0.1019
Gardelegen	0.0095	0.2091
Großenkneten	0.0561	0.1851
Kiefersfelden-Gach	0.0572	0.2602
Kleve	0.0647	0.1692
Müncheberg	0.0197	0.2252
Nienburg	0.0294	0.1381
Rotenburg (Wümme)	0.0069	0.0725
Rothenburg ob der Tauber	0.0077	0.2274
Ueckermünde	0.0498	0.0987
Waren (Müritz)	0.0213	0.1366
average	0.0275	0.1427
